# C-reactive protein in traditional melanesians on Kitava

**DOI:** 10.1186/s12872-020-01812-7

**Published:** 2020-12-17

**Authors:** Pedro Carrera-Bastos, Maelán Fontes-Villalba, Michael Gurven, Frits A. J. Muskiet, Torbjörn Åkerfeldt, Ulf Lindblad, Lennart Råstam, Johan Frostegård, Yinon Shapira, Yehuda Shoenfeld, Yvonne Granfeldt, Kristina Sundquist, Tommy Jönsson

**Affiliations:** 1 Center for Primary Health Care Research, Lund University/Region Skåne, Skåne University Hospital, Jan Waldenströms gata 35, CRC, hus 28 plan 11, 205 02 Malmö, Sweden; 2 Department of Anthropology, University of California, Santa Barbara, USA; 3 Department of Laboratory Medicine, University Medical Centre Groningen, University of Groningen, Groningen, Netherlands; 4 Department of Medical Sciences, Uppsala University, Uppsala University Hospital, Uppsala, Sweden; 5 School of Public Health and Community Medicine, University of Gothenburg, Gothenburg, Sweden; 6 Department of Clinical Sciences, Community Medicine, Lund University, Lund, Sweden; 7 IMM, Unit of Immunology and Chronic Disease, Karolinska Institutet, Stockholm, Sweden; 8 Department of Ophthalmology, Rambam Health Care Campus, Haifa, Israel; 9 Zabludowicz Center for Autoimmune Diseases, Sheba Medical Center (Affiliated To Tel-Aviv University), Tel-Hashomer, Israel; 10 Sackler Faculty of Medicine, Tel-Aviv University, Tel-Hashomer, Israel; 11 I.M. Sechenov First Moscow State Medical University of the Ministry of Health of the Russian Federation (Sechenov University), Saint Petersburg, Russia; 12 Department of Food Technology, Engineering and Nutrition, Lund University, Lund, Sweden

**Keywords:** C-reactive protein, Cardiovascular risk, Traditional melanesians

## Abstract

**Background:**

Population-based levels of the chronic low-grade systemic inflammation biomarker, C-reactive protein (CRP), vary widely among traditional populations, despite their apparent absence of chronic conditions associated with chronic low-grade systemic inflammation, such as type 2 diabetes, metabolic syndrome and cardiovascular disease. We have previously reported an apparent absence of aforementioned conditions amongst the traditional Melanesian horticulturalists of Kitava, Trobriand Islands, Papua New Guinea. Our objective in this study was to clarify associations between chronic low-grade systemic inflammation and chronic cardiometabolic conditions by measuring CRP in a Kitava population sample. For comparison purposes, CRP was also measured in Swedish controls matched for age and gender.

**Methods:**

Fasting levels of serum CRP were measured cross-sectionally in ≥ 40-year-old Kitavans (*N* = 79) and Swedish controls (*N* = 83).

**Results:**

CRP was lower for Kitavans compared to Swedish controls (*Mdn* 0.5 mg/L range 0.1—48 mg/L and *Mdn* 1.1 mg/L range 0.1—33 mg/L, respectively, *r* = .18 *p* = .02). Among Kitavans, there were small negative associations between lnCRP for CRP values < 10 and total, low-density lipoprotein (LDL) and non-high-density lipoprotein (non-HDL) cholesterol. Among Swedish controls, associations of lnCRP for CRP values < 10 were medium positive with weight, body mass index, waist circumference, hip circumference and waist-hip ratio and low positive with triglyceride, total cholesterol-HDL cholesterol ratio, triglyceride-HDL cholesterol ratio and serum insulin.

**Conclusions:**

Chronic low-grade systemic inflammation, measured as CRP, was lower among Kitavans compared to Swedish controls, indicating a lower and average cardiovascular risk, respectively, for these populations.

## Background

Chronic non-communicable conditions such as metabolic syndrome, type 2 diabetes and cardiovascular disease are associated with chronic low-grade systemic inflammation [1–5]. The causality of this association remains unresolved; this is possibly because there is no consensus as to which markers best represent chronic low-grade systemic inflammation [6–8]. A commonly used marker is C-reactive protein (CRP) [8, 9]. CRP adds prognostic information on cardiovascular risk comparable to blood pressure or cholesterol, where circulating levels < 1 (lower), 1 to 3 (average), and > 3 mg/L (higher) indicate relative cardiovascular risk, whereas values above 10 mg/L typically reflect acute inflammatory responses, such as those induced by infections or trauma [9]. Given the association between the aforementioned conditions and chronic low-grade systemic inflammation, we hypothesized that CRP would be low in traditional populations considered minimally afflicted by those conditions. However, CRP levels from such populations vary from low to high. For example, low values reported for Shuar forager-horticulturalists of the Ecuadorian Amazon with a median CRP of 0.5 mg/L and the subsistence-agriculturalists from rural Ghana with a median CRP of 0.8 mg/L, but with higher values for Hadza hunter-gatherers from Tanzania with a median CRP of 1.5 mg/L and Tsimane forager-horticulturalists of Bolivia with a median CRP just below 3 mg/L [10–13]. In this study we report complementary results on CRP from the Melanesian population of Kitava, Trobriand Islands, Papua New Guinea, among whom we have previously reported an apparent absence of metabolic syndrome, type 2 diabetes and cardiovascular disease [14–23].

For comparison purposes, we also report CRP results from an age and sex-matched Swedish control population. Our previous comparison of these populations revealed higher levels of Kitavan anti-phosphorylcholine (anti-PC) antibodies [21, 23], for which anti-inflammatory mechanisms have been suggested to mediate a reported inverse association with cardiovascular disease [24, 25]. Higher levels of Kitavan anti-PC antibodies could be caused by prior infection and autoimmune processes [22, 26].

## Methods

### Ethics statement

Kitava: The study was approved by the Research Ethical Committee of the Medical Faculty at Lund University (LU 246-96), the Medical Research Advisory Committee of Papua New Guinea, other Papua New Guinea national and provincial bodies and at the community level by the inhabitants and their tribal leaders. Informed consent through personal contact was obtained from all participants.

Sweden: The ethics committee of the Medical Faculty at Göteborg University approved the study protocol (GU 343-93), and all participants gave informed consent.

### Study populations & data collection

Kitava: The Kitava study was conducted in 1990 as a cross-sectional study of a partly random and partly self-selected cohort among the 2250 inhabitants of the island of Kitava, Trobriand Islands, Papua New Guinea [14, 15]. Clinical observations, semi-structured interviews (N = 213), electrocardiograms (N = 171), clinical examinations (N = 203) and laboratory measurements (N = 166) revealed an absence of metabolic syndrome, type 2 diabetes and cardiovascular disease [14–23]. Ages were calculated from well-known historical events and were considered accurate to within three years for most subjects. At the time of study, the Kitavans were horticulturalists (i.e. traditional farmers of tubers and fruits) and dietary staples were cultivated tubers (mainly yam, sweet potato and taro, but also small amounts of cassava), supplemented by fruits, leaves, nuts (including coconut), fish, maize and beans [14]. Electricity, telephones and motor vehicles were absent, and the average level of physical activity (PAL) was 1.7 multiples of the basal metabolic rate, which is classified as moderate-to-active [19]. The prevalence of smoking and betel chewing among adults was 76% and 100%, respectively [14, 15], and serology indicated a high parasitic and infectious burden [21–23]. For this study, all subjects older than 50 years (*N* = 206) and 10% of those aged 40–50 (*n* = 41) were eligible. The acceptance rate for serum sampling was only 41% and self-selected subjects aged 40–50 years were therefore also included (Additional file 1: Figure S1). The latter consisted of persons excluded by the random generator but willing to participate. Individual subjects did not receive payment for their participation. In the age group older than 80 years, non-participants were slightly more often disabled and older than participants, but non-participants in other age groups did not differ from participants in body composition, agility or level of physical activity [27]. The Kitavans excluded from this study due to having declined serum sampling were more likely to be female and to have lower anthropometrics, but also higher systolic blood pressure compared to included Kitavans (Additional file 1: Figure S1 and Table S1). The included self-selected Kitavans had lower leptin but were heavier and, as expected, also younger compared to the randomly sampled Kitavans (Additional file 1: Figure S1 and Table S2).

Serum samples were thus obtained from 110 Kitavan subjects, aged 40–86 years before 9 a.m., after an overnight fast abstaining from eating, smoking and betel chewing for at least nine hours. The samples were centrifuged within three hours and then frozen within 60 min and transported at − 130 °C to Sweden and subsequent storage at -70 °C until analysis.

Sweden: The Skara Population Study was conducted in 1993–94 as an age- and sex-stratified, random, cross-sectional sample from subjects ≥ 40-years-old living in the small town of Skara or its surrounding countryside (age, gender distribution and morbidity for county is similar to the general Swedish population [28]), who were invited to the healthcare center for a health examination similar to the extended annual check-up of patients with hypertension and type 2 diabetes [29, 30]. From each 10-year age-category, between 40 and 79 years of age, a total of 150 male and 150 female residents were invited. In the ages 80 years and above, 100 male and 100 female subjects were also invited. Of these 1400 invited subjects, 1109 (80%) accepted the invitation and completed a physical examination. Blood specimens were drawn in the morning after an overnight fast abstaining from eating and smoking and immediately stored at -80 °C until analysis. For the present study, Skara participants were randomly selected to match by age and sex each of the 110 Kitavan subjects. Prior comparison of Kitavans with these Swedish controls indicated a more atherogenic risk factor profile among Swedes compared to Kitavans, although smoking was lower at 25% [21]. Among these 110 Swedish controls, a diagnosis of type 2 diabetes (*n* = 12) and/or hypertension (*n* = 15) was present in 24 participants, which was, respectively, roughly two thirds higher and two thirds lower than expected based on contemporary local and national prevalence of these conditions [28, 31–34]. These diagnoses were either determined by the subjects’ own personal physician, which was the case for 20 participants (*n* = 11 for previous diagnosis for both type 2 diabetes and/or hypertension), or during the study examination. Swedish controls with sample analyzed for CRP without a diagnosis of type 2 diabetes or hypertension were taller and, as expected, younger with lower systolic blood pressure, fasting blood glucose, triglyceride, total cholesterol-HDL cholesterol ratio and triglyceride-HDL cholesterol ratio compared to Swedish controls with sample analyzed for CRP with a diagnosis of type 2 diabetes or hypertension (Additional file 1: Table S4).

In both populations, standard methods were used for measurements of traditional risk factors, as described previously [15, 21, 35]. In Kitava, the blood pressure was measured with subjects in the sitting position after a few minutes rest and the upper arm kept parallel to the sternum, and in the Swedish controls the blood pressure was measured after five minutes rest in a supine position with the upper arm at heart level, which should result in lower blood pressure among the latter [36]. Anti-PC antibodies were determined by enzyme-linked immunosorbent assay, as described previously [21, 23]. The Kitavan samples were previously also tested for presence of antinuclear antibodies and autoantibodies associated with thrombophilia, vasculitis, and gastrointestinal disease, as well as antibodies against infectious agents (i.e. Epstein-Barr virus, cytomegalovirus, Treponema pallidum and Toxoplasma gondii) [22, 26].

### Measurements of CRP

Serum CRP analysis was performed in 2007 in an accredited laboratory using a turbidimetric assay with the same reagent lot for both populations on an Architect Ci8200 analyzer (Abbott Laboratories, Abbott Park, IL, USA). The CRP assay had a limit of quantification of 0.20 mg/L and a total coefficient of variation of 4% at 1.4 mg/L. As per assay specifications, measurements below 0.20 mg/L were considered uncertain, and were here replaced with 0.20 mg/L divided by the square root of 2 (14 Kitavans and 8 Swedish controls) [37]. The CRP assay used is a high-sensitivity method with a limit of quantification slightly higher and a coefficient of variation slightly lower than for the CRP assays used in the Shuar of Ecuador (0.028 mg/L) [10], rural Ghanaians (0.1 mg/L) [11], Hadza of Tanzania (0.028 mg/L) [12] and Tsimane of Bolivia (0.1 mg/L) [13]. 58 samples were lost to analysis due to missing and insufficient samples. CRP measurements were therefore only possible in 79 Kitavans and 83 Swedish controls, which constituted 59 matched pairs of Kitavans and Swedish controls and another 20 Kitavans and 24 Swedish controls without their respective matched counterpart (Additional file 1: Figure S1). Swedish controls with samples analyzed for CRP had lower triglyceride and triglyceride-HDL cholesterol ratio compared to Swedish controls with samples lost to analysis (Additional file 1: Table S3). Kitavans with samples analyzed for CRP were younger and relatively fewer females and smokers and had lower triceps skinfold thickness and leptin concentration compared to Kitavans with samples lost to analysis (Additional file 1: Table S3-4). Kitavans with samples analyzed for CRP were relatively more likely to be smokers, lighter, shorter, slimmer and with lower blood pressure, glucose, insulin and cholesterol (total, HDL, LDL and non-HDL) compared to Swedish controls with samples analyzed for CRP (Table 1).

**Table 1:** Clinical Characteristics and C-reactive Protein of Kitavans and Swedish Controls with Samples Analyzed for C-reactive Protein

Variable	Kitavans		Swedish controls		*r*
Male / female, *n* (%)	61 (77%) / 18 (23%)		58 (70%) / 25 (30%)		
Smoker / non-smoker, *n* (%)**	52 (69%) / 23 (31%)		20 (24%) / 63 (76%)		0.45
Age, years *M (SD)*	58 (11)		60 (10)		
Weight, kg M (SD)**	48 (8)		77 (13)		0.83
Height, cm M (SD)**	159 (7)		173 (9)		0.65
Body mass index, kg/m^2^ M (SD)**	19 (2)		26 (4)		0.78
Waist circumference, cm M (SD)**	73 (5)		89 (12)		0.73
Hip circumference, cm M (SD)**	78 (5)		101 (8)		0.86
Waist-hip ratio M(SD)**	0.9 (0.0)		0.9 (0.1)		0.30
Systolic blood pressure, mm Hg M (SD)**	117 (17)		136 (19)		0.47
Diastolic blood pressure, mm Hg M (SD)**	70 (7)		77 (9)		0.42
Fasting blood glucose, mmol/L M (SD)**	3.8 (0.7)		5.1 (1.3)		0.64
Fasting serum insulin, IU/mL M (SD)**	4.7 (5.2)		6.2 (6.1)		0.28
Total cholesterol, mmol/L M (SD)**	5.0 (1.2)		5.7 (1.0)		0.31
LDL, mmol/L M (SD)**	3.4 (1.1)		4.0 (0.9)		0.33
HDL, mmol/L M (SD)*	1.0 (0.3)		1.2 (0.3)		0.18
Triglyceride, mmol/L *M (SD)*	1.2 (0.5)		1.3 (0.8)		
Non-HDL cholesterol, mmol/L M (SD)**	4.0 (1.1)		4.6 (0.9)		0.28
Total cholesterol-HDL ratio, mol/mol *M (SD)*^a^	5.0 (1.4)		5.2 (1.4)		
LDL-HDL ratio, mol/mol *M (SD)*^a^	3.4 (1.3)		3.6 (1.1)		
Triglyceride-HDL ratio, mol/mol *M (SD)*^a^	1.3 (0.7)		1.3 (1.1)		
C-reactive protein, mg/L *Mdn* (range)*	0.47 (0.14–48)		1.10 (0.14–33)		0.18
C-reactive protein < 10, mg/L *Mdn* (range)*	0.45 (0.14–8.2)		0.96 (0.14–7.2)		0.21

*Note.* LDL = low-density lipoprotein cholesterol. HDL = high-density lipoprotein cholesterol. ^a^ Values in mmol/L for ratios. * and ** *p* < .05 and *p* < .001, respectively

### Statistical analysis

For group comparisons the Student’s *t* test, the Mann–Whitney *U* test and the *X*^2^ test were used, as appropriate. The group distributions for the natural logarithm of CRP (lnCRP) were visually reasonably normal. For estimation of correlation between variables the Pearson correlation was used. For estimations of linear associations between independent variables and chronic low-grade systemic inflammation, simple and multiple linear regression was used with lnCRP for CRP values below 10 as dependent. When simple linear regression was not possible, the Spearman rank test was used instead. An alpha level of 0.05 was used for all statistical tests.

## Results

**Fig. 1:**
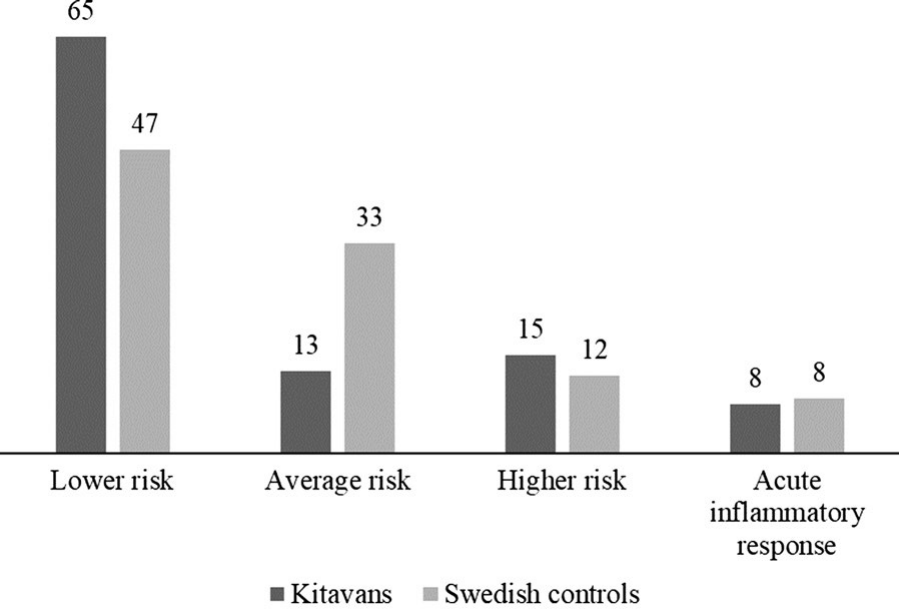
C-reactive Protein Values and Cardiovascular Risk Levels. *Note.* Above each bar is displayed the corresponding percentage (%) of Kitavans and Swedish controls with C-reactive protein (CRP) values indicating lower (CRP < 1 mg/L), average (CRP 1–3 mg/L) and higher (CRP 3–10 mg/L) relative cardiovascular risk, or acute inflammatory response, such as those induced by infections or trauma (CRP > 10 mg/L)

There were small group differences in CRP between Kitavans and Swedish controls for all CRP values and for CRP values below 10, with lower CRP for Kitavans (Fig. 1 and Table 1). There was no group difference in CRP between self-selected and not self-selected, male or female and smoking or non-smoking Kitavans, nor between male or female and smoking or non-smoking Swedish controls and Swedish controls with or without a diagnosis of type 2 diabetes and/or hypertension (Additional file 1: Table S2-4). In a sensitivity analysis, when removing all 19 participants with a diagnosis of type 2 diabetes (*n* = 10) and/or hypertension (*n* = 11) for which CRP was analyzed, the small group difference in CRP for all CRP values disappeared (*p* = 0.065) but the small group difference in CRP for CRP values below 10 remained (*r* = 0.18, *p* = 0.038).

Among Kitavans, the proportion of participants with CRP values below or equal to the CRP median and CRP values above the CRP median but below 10 did not differ by serology (positive or negative) for antitoxoplasma or anti-Epstein-Barr virus (EBV)-early Ag (EA) antibodies. The remaining anti-infective agent antibodies and autoimmunity antibodies had too few either positive or negative serology values to perform a valid *X*^2^ test. Instead, in a Mann–Whitney *U* test, serological variables analyzable for differences in CRP values below 10 were antitoxoplasma, anti-EBV-viral capsid antigen (VCA), anti-EBV-EA, anti-EBV-nuclear antigen (NA), antitreponemal, anti-glomerular basement membrane (GBM), anti-double-stranded DNA (dsDNA), antichromatin, anti-ribonucleoprotein-A (RNP-A), anti-Scl-70, anti-Smith, anticentromere B and anti-RNP. Of these, only anticentromere B serology displayed a difference in CRP, which was medium higher among centromere B positive participants (*n* = 4) compared to centromere B negative participants (*n* = 63) (*Mdn* 3.1 and 0.4 and range 1.8–4.6 and 0.1–8.2, respectively, *r* = 0.30, *p* = 0.02). There was no association between lnCRP for CRP values below 10 and anti-PC antibodies among either Kitavans or Swedish controls, nor among all participants, with data from Kitavans and Swedish controls combined.

Among Kitavans, there were small negative associations between lnCRP for CRP values below 10 and total, LDL and non-HDL cholesterol (*r* = −0.25, −0.24 and −0.23, respectively, *B* = −0.28, −0.28 and −0.26, *p* = 0.031, 0.045 and 0.050, respectively), and no association between lnCRP for CRP values below 10 and triceps skinfold and leptin.

Among Swedish controls, the associations of lnCRP for CRP values below 10 were medium positive with weight, body mass index, waist circumference, hip circumference and waist-hip ratio (*r* = 0.40, 0.47 0.46, 0.40 and 0.31, respectively, *B* = 0.031, 0.11, 0.039, 0.050 and 3.8, respectively, *p* < 0.001 for all except waist-hip ratio for which *p* = 0.0062) and low positive with triglyceride, total cholesterol-HDL cholesterol ratio, triglyceride-HDL cholesterol ratio and serum insulin (*r* = 0.26, 0.29, 0.27 and 0.23, *p* = 0.023, 0.010, 0.020 and 0.042, respectively).

Among all participants, with data from Kitavans and Swedish controls combined, there were small to medium positive associations in linear regression between lnCRP for CRP values below 10 and weight, body mass index and waist and hip circumference (*r* = 0.26, 0.31, 0.33 and 0.26, respectively, *B* = 0.02, 0.08, 0.03 and 0.02, respectively, *p* = 0.0018, < 0.0001, < 0.0001 and 0.0014, respectively). Weight, body mass index, waist circumference and hip circumference were all highly correlated (*r*_*s*_ > 0.90 for all), as expected. There were also small positive associations between lnCRP for CRP values below 10 and the grouping variable for Kitavan and Swedish control groups and fasting blood glucose (*r* = 0.18 and *r*_*s*_ = 0.22, respectively, *B* = 0.41 for the grouping variable, *p* = 0.029 and 0.0070, respectively). Only waist circumference was included in the model when entered together with the grouping variable for Kitavan and Swedish control groups and fasting blood glucose as predictors in a stepwise multiple linear regression model with lnCRP for CRP values below 10 as dependent.

## Discussion

We found a low median CRP of 0.5 mg/L for the Kitavans, which may indicate for this population a low prevalence of chronic low-grade systemic inflammation and a corresponding lower cardiovascular risk [9]. A lower cardiovascular risk for the Kitavans agree with our previous reports on their absence of cardiometabolic disorders [14–23]. The results also concur with findings of low CRP among the Shuar of Ecuador and rural Ghanaians [10, 11]. However, the low Kitavan CRP contrasts with the higher CRP levels among the Hadza of Tanzania and the Tsimane of Bolivia [12, 13]. The higher CRP among the latter populations was attributed to a high parasitic and infectious burden [13], but the low Kitavan CRP does not agree with such an interpretation, since previously reported serology also suggested a high parasitic and infectious burden among the Kitavans [22, 26]. The low CRP among the physically active [19] but mostly smoking [15] Kitavans with a non-western like dietary pattern [14, 16, 18] agrees with previously reported associations for CRP with physical inactivity [38, 39] and western-like dietary patterns [40], but contradicts previously reported associations with smoking [41–43]. The latter disagreement is compounded by the lack of group difference in CRP between smokers and non-smokers in both populations, although it is conceivable that some quantitative estimate of smoking intensity and duration would be needed to reveal such associations.

CRP was higher among the four Kitavans with positive serology for anti-centromere B antibodies, which can be found in a variety of connective tissue diseases (e.g. CREST syndrome [calcinosis, Raynaud's phenomenon, esophageal dysmotility, sclerodactyly, telangiectasia], diffuse systemic sclerosis, primary biliary cirrhosis) but also in patients without apparent connective tissue disease [44]. As reported previously, the Kitavans have a high proportion of positive serology for anti-nuclear antibodies (ANA), of which anti-centromere B antibodies is one, which could indicate a true high risk and prevalence of autoimmune diseases, but which also could be due to induction of autoantibodies by the high parasitic and infectious burden among Kitavans [26]. The higher CRP among the four anti-centromere B positive Kitavans could thus signal inflammatory activity in an associated autoimmune disease but could also signal inflammatory activity in an anti-centromere B autoantibody-inducing infectious disease. Either signal could indicate support for a causal link with higher levels of Kitavan anti-PC antibodies, albeit weak and ambiguous. The lack of association between CRP and anti-PC antibodies does not support an anti-inflammatory mechanism behind the latter’s inverse association with cardiovascular disease [24, 25].

The Kitavan CRP is lower than the median CRP of 1.1 mg/L for the Swedish controls, which is in line with an average to lower cardiovascular risk for the latter [45]. The lower Kitavan CRP also agrees with a less atherogenic risk factor profile in clinical characteristics compared to the Swedish controls, except for smoking. The medium associations found for CRP with adiposity measures among the Swedish controls agree with previous epidemiological findings [1], but the lack of association among the Kitavans is noteworthy, although the lower variation in adiposity measures among Kitavans could make such an association less discernable. Lack of association between CRP and adiposity measures was also found for the subsistence-agriculturalists from rural Ghana, with a low mean body mass index (BMI) of 18.4 kg/m^2^ [11], which is comparable to the low mean BMI of 18.9 kg/m^2^ among the Kitavans, suggesting that CRP may not be discernably associated with measures of adiposity in lean traditional populations. Accordingly, CRP was associated with BMI among the Shuar with a mean BMI of almost 26 kg/m^2^ [10]. Furthermore, epidemiological genetic studies [46] and CRP reductions following weight loss in intervention studies [47] indicate that CRP is a marker of adiposity rather than the other way around. In lean populations, such as the Kitavans, the association between CRP and adiposity measures thus seems to be non-existent or non-discernable. Consistently, in association analysis among both groups combined, the grouping variable and fasting blood glucose, both for which there were small associations with CRP, were excluded as not significantly contributing to the variation in CRP when analyzed together with waist circumference, which was strongly correlated with other adiposity measures. The group difference in CRP between Kitavans and Swedish controls thus seems to be due to group differences in adiposity.

In addition to a lack of association with adiposity measures, the Kitavan CRP was also negatively associated with total, non-HDL and LDL cholesterol, which is opposite to associations found in both healthy controls and patients with cardiometabolic disease [48–50], but in accordance with findings from the Tsimane of Bolivia [51]. In contrast, the CRP of the Swedish controls was positively associated with total cholesterol-HDL cholesterol ratio, triglyceride, triglyceride-HDL cholesterol ratio and serum insulin, in line with the putative role of inflammation in dyslipidemia [2, 52, 53] and insulin resistance [54, 55], further suggesting differences between lean traditional populations and populations with a more atherogenic health profile when evaluating CRP associations.

## Limitations

The small size of the study populations impairs the ability of the study to detect potential differences between groups and associations within groups. The planned size of the Kitavan study population, albeit small, was deemed sufficient to assess the non-CRP containing cardiovascular risk factors for which the study was originally designed. The resulting Kitavan population size was further limited by recruitment difficulties, and, for the analysis of CRP, also by samples lost to analysis.

The slightly higher limit of quantification of the CRP assay used in this study, as compared to the CRP assays used in the referenced studies on CRP in other traditional populations, impairs the ability of the study to discern individual differences in low levels of chronic inflammation. However, a lower limit of quantification can also adversely affect the efficiency and reproducibility of the assay. The coefficient of variation of the CRP assay used in this study was lower, and thus more precise and reproducible, than the coefficients of variation of the CRP assays used in the referenced studies on CRP in other traditional populations. We believe the slightly higher limit of quantification of the CRP assay used in our study is an acceptable trade-off for higher precision and reproducibility and should not affect the conclusions of the study.

Each subject in this study only have one sample for analysis and thus only one resulting CRP value. More samples per subject would have been needed to better establish an accurate level of chronic low-grade systemic inflammation for each subject and population, since a single higher CRP value could be due to intra-individual variation from acute stress or infections [9]. However, requiring more samples per subject could also have negatively affected the already difficult recruitment for the study.

## Conclusions

In conclusion, chronic low-grade systemic inflammation, measured as CRP, was lower among Kitavans compared to Swedish controls, indicating a lower and average cardiovascular risk, respectively, for these populations.

## Supplementary information

**Additional file 1.** Enrollment, Samples Analyzed for C-reactive Protein, and Clinical Characteristics of Subgroups.

## Data Availability

The dataset analyzed during the current study are available from the corresponding author on reasonable request.

## Abbreviations

CRP: C-reactive protein
LDL: Low-density lipoprotein
non-HDL: Non-high-density lipoprotein
anti-PC: Anti-phosphorylcholine
PAL: Level of physical activity
lnCRP: Natural logarithm of CRP
EBV: Epstein-Barr virus
EA: Early Ag
VCA: Viral capsid antigen
NA: Nuclear antigen
GBM: Glomerular basement membrane
dsDNA: Double-stranded DNA
RNP-A: Ribonucleoprotein-A
CREST syndrome: Calcinosis, Raynaud's phenomenon, esophageal dysmotility, sclerodactyly, telangiectasia
ANA: Anti-nuclear antibodies
