# Supplementary material for: C-reactive protein in traditional melanesians on Kitava
Source: BMC Cardiovasc Disord. 2020 Dec 17;20:524. doi: 10.1186/s12872-020-01812-7 (PMC7745357; doi:10.1186/s12872-020-01812-7)
Supplement: Supplementary file 1 — Additional file 1. Enrollment, Samples Analyzed for C-reactive Protein, and Clinical Characteristics of Subgroups. [file 12872_2020_1812_MOESM1_ESM.pdf]

Figure S1

Enrollment and Samples Analyzed for C-reactive Protein

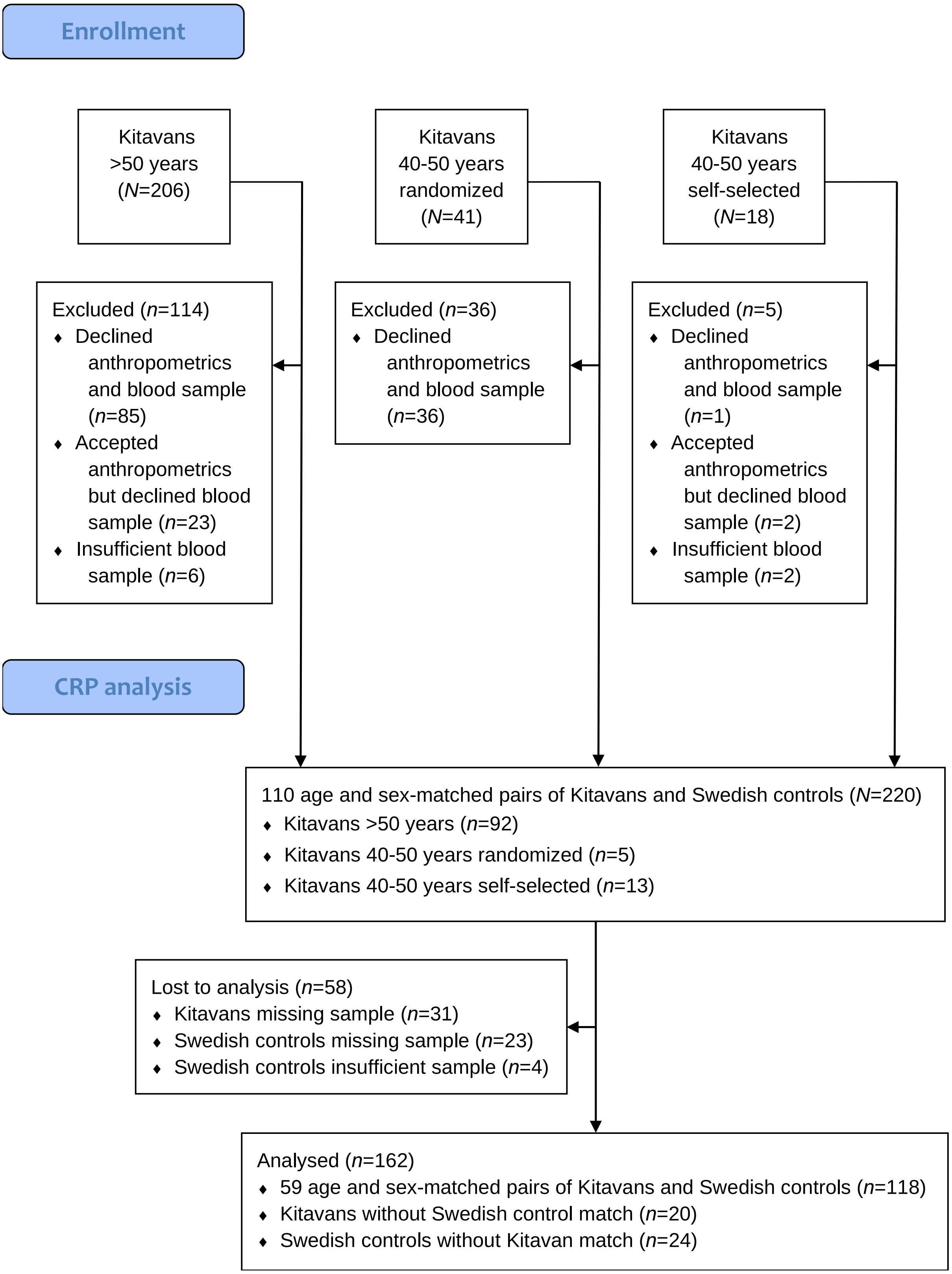

Table S1

Clinical Characteristics of Included and Excluded Kitavans with Anthropometrics

Variable	Included	Excluded
Male / female, *n* (%)*	78 (71%) / 32 (29%)	16 (48%) / 17 (52%)
Smoker / non-smoker, *n* (%)	79 (76%) / 25 (24%)	25 (89%) / 3 (11%)
Age, years *M (SD)*	60 (11)	58 (10)
Weight, kg *M (SD)**	48 (8)	43 (8)
Height, cm *M (SD)* *	158 (7)	154 (7)
Body mass index, kg/m^2 *M (SD)* *	19 (2)	18 (2)
Waist circumference, cm *M (SD)* *	73 (5)	69 (6)
Hip circumference, cm *M (SD)* *	79 (5)	76 (6)
Waist-hip ratio *M(SD)*	0.9 (0.0)	0.9 (0.1)
Triceps skinfold, mm *M (SD)*	7.6 (2.8)	8.4 (3.4)
Systolic blood pressure, mm Hg *M (SD)* *	118 (18)	124 (15)
Diastolic blood pressure, mm Hg *M (SD)*	70 (7)	72 (7)

Note. * $p < .05$.

Table S2

Clinical Characteristics of Included not Self-selected and Self-selected Kitavans

Variable	Not self-selected	Self-selected
Male / female, *n* (%)	67 (69%) / 30 (31%)	11 (85%) / 2 (15%)
Smoker / non-smoker, *n* (%)	72 (78%) / 20 (22%)	7 (58%) / 5 (42%)
Age, years *M (SD)* *	62 (9)	44 (3)
Weight, kg *M (SD)**	47 (7)	52 (7)
Height, cm *M (SD)*	158 (7)	158 (4)
Body mass index, kg/m^2 *M (SD)* *	19 (2)	21 (2)
Waist circumference, cm *M (SD)*	72 (5)	74 (5)
Hip circumference, cm *M (SD)*	79 (5)	79 (4)
Waist-hip ratio *M(SD)*	0.9 (0.0)	0.9 (0.1)
Triceps skinfold, mm *M (SD)*	7.6 (2.8)	7.3 (3.4)
Systolic blood pressure, mm Hg *M (SD)*	118 (18)	118 (13)
Diastolic blood pressure, mm Hg *M (SD)*	71 (72)	71 (8)
Fasting blood glucose, mmol/L *M (SD)*	3.9 (0.7)	3.6 (0.5)
Fasting serum insulin, IU/mL *M (SD)*	3.8 (3.1)	8.8 (9.9)
Total cholesterol, mmol/L *M (SD)*	5.1 (1.2)	4.9 (1.1)
LDL, mmol/L *M (SD)*	3.5 (1.1)	3.3 (1.0)
HDL, mmol/L *M (SD)*	1.1 (0.3)	1.1 (0.2)
Triglyceride, mmol/L *M (SD)*	1.2 (0.4)	1.2 (0.6)
Non-HDL cholesterol, mmol/L *M (SD)*	4.0 (1.1)	3.8 (1.1)
Total cholesterol-HDL ratio, mol/mol *M (SD)* [a]	5.0 (1.4)	4.7 (1.3)
LDL-HDL ratio, mol/mol *M (SD)* [a]	3.4 (1.2)	3.1 (1.2)
Triglyceride-HDL ratio, mol/mol *M (SD)* [a]	1.2 (0.6)	1.2 (0.7)
Leptin, ng/mL *Mdn* (range)*	1.9 (0.7 - 25)	1.4 (0.8 - 8.8)
C-reactive protein, mg/L *Mdn* (range)	0.47 (0.14 - 48)	0.46 (0.14 - 6.9)

Note. LDL = low-density lipoprotein cholesterol. HDL = high-density lipoprotein cholesterol.

[a] Values in mmol/L for ratios. * $p < .05$.

Table S3

Clinical Characteristics of Kitavans and Swedish Controls with Samples Analyzed for C-reactive Protein and Samples Lost to Analysis

	Kitavans		Swedish controls	
Variable	Samples analysed for CRP	Samples lost to analysis	Samples analysed for CRP	Samples lost to analysis
Male / female, *n* (%)*	61 (77%) / 18 (23%)	17 (55%) / 14 (45%)	58 (70%) / 25 (30%)	20 (74%) / 7 (26%)
Smoker / non-smoker, *n* (%)*	52 (69%) / 23 (31%)	27 (93%) / 2 (7%)	20 (24%) / 63 (76%)	7 (26%) / 20 (74%)
Diabetic / non-diabetic, *n* (%)	0 (0%) / 79 (100%)	0 (0%) / 79 (100%)	10 (12%) / 73 (88%)	2 (7%) / 25 (93%)
Hypertensive / non-hypertensive, *n* (%)	0 (0%) / 79 (100%)	0 (0%) / 79 (100%)	11 (13%) / 72 (87%)	4 (15%) / 23 (85%)
Age, years *M (SD)* *	58 (11)	64 (8)	60 (10)	59 (12)
Weight, kg *M (SD)*	48 (8)	46 (6)	77 (13)	80 (13)
Height, cm *M (SD)*	159 (7)	156 (6)	173 (9)	174 (8)
Body mass index, kg/m^2 *M (SD)*	19 (2)	19 (2)	26 (4)	26 (4)
Waist circumference, cm *M (SD)*	73 (5)	73 (4)	89 (12)	92 (11)
Hip circumference, cm *M (SD)*	78 (5)	79 (5)	101 (8)	102 (9)
Waist-hip ratio *M(SD)*	0.9 (0.0)	0.9 (0.0)	0.9 (0.1)	0.9 (0.1)
Triceps skinfold, mm *M (SD)* *	7.3 (2.6)	8.6 (3.3)	No data	No data
Systolic blood pressure, mm Hg *M (SD)*	117 (17)	122 (20)	136 (19)	132 (13)
Diastolic blood pressure, mm Hg *M (SD)*	70 (7)	71 (6)	77 (9)	77 (11)
Fasting blood glucose, mmol/L *M (SD)*	3.8 (0.7)	4.0 (0.5)	5.1 (1.4)	4.8 (0.8)
Fasting serum insulin, IU/mL *M (SD)*	4.7 (5.2)	3.6 (2.8)	6.2 (6.1)	5.6 (4.2)
Total cholesterol, mmol/L *M (SD)*	5.0 (1.2)	5.1 (1.1)	5.7 (1.0)	6.0 (0.8)
LDL, mmol/L *M (SD)*	3.4 (1.1)	3.5 (1.0)	4.0 (0.9)	4.2 (0.7)
HDL, mmol/L *M (SD)*	1.0 (0.3)	1.1 (0.2)	1.2 (0.3)	1.1 (0.2)
Triglyceride, mmol/L *M (SD)* **	1.2 (0.5)	1.1 (0.4)	1.3 (0.8)	1.5 (0.6)
Non-HDL cholesterol, mmol/L *M (SD)*	4.0 (1.1)	4.0 (1.1)	4.6 (0.9)	4.9 (0.8)
Total cholesterol-HDL ratio, mol/mol *M (SD)* [a]	5.0 (1.4)	4.8 (1.4)	5.2 (1.4)	5.7 (1.2)
LDL-HDL ratio, mol/mol *M (SD)* [a]	3.4 (1.3)	3.3 (1.2)	3.6 (1.1)	4.0 (1.0)
Triglyceride-HDL ratio, mol/mol *M (SD)* [a]**	1.3 (0.7)	1.1 (0.6)	1.3 (1.1)	1.4 (0.7)
Leptin, ng/mL *Mdn* (range)*	1.7 (0.7 - 25)	2.2 (0.9 - 8.7)	No data	No data

Note. LDL = low-density lipoprotein cholesterol. HDL = high-density lipoprotein cholesterol. [a] Values in mmol/L for ratios. * and ** *p* < .05 for group comparison among Kitavans and Swedish controls, respectively.

Table S4

Clinical Characteristics of Swedish Controls with Samples Analyzed for C-reactive Protein Without or With a Diagnose of Type 2 Diabetes or Hypertension

Variable	Without diagnose	With diagnose
Male / female, *n* (%)	44 (69%) / 20 (31%)	14 (74%) / 5 (26%)
Smoker / non-smoker, *n* (%)	17 (27%) / 47 (73%)	3 (16%) / 16 (84%)
Age, years *M (SD)* *	58 (10)	66 (10)
Weight, kg *M (SD)*	76 (13)	78 (15)
Height, cm *M (SD)* *	174 (9)	169 (8)
Body mass index, kg/m^2 *M (SD)*	25 (3)	27 (6)
Waist circumference, cm *M (SD)*	88 (11)	94 (14)
Hip circumference, cm *M (SD)*	101 (7)	103 (12)
Waist-hip ratio *M(SD)*	0.9 (0.1)	0.9 (0.1)
Systolic blood pressure, mm Hg *M (SD)* *	134 (20)	145 (15)
Diastolic blood pressure, mm Hg *M (SD)*	77 (9)	79 (9)
Fasting blood glucose, mmol/L *M (SD)* *	4.8 (0.6)	6.1 (2.6)
Fasting serum insulin, IU/mL *M (SD)*	5.6 (5.4)	8.1 (7.9)
Total cholesterol, mmol/L *M (SD)*	5.7 (1.0)	5.8 (0.8)
LDL, mmol/L *M (SD)*	4.0 (1.0)	4.1 (0.8)
HDL, mmol/L *M (SD)*	1.2 (0.3)	1.1 (0.3)
Triglyceride, mmol/L *M (SD)* *	1.2 (0.7)	1.6 (0.9)
Non-HDL cholesterol, mmol/L *M (SD)*	4.5 (1.0)	4.8 (0.8)
Total cholesterol-HDL ratio, mol/mol *M (SD)* [a]*	5.0 (1.3)	5.8 (1.5)
LDL-HDL ratio, mol/mol *M (SD)* [a]	3.5 (1.1)	4.0 (1.2)
Triglyceride-HDL ratio, mol/mol *M (SD)* [a]*	1.1 (0.9)	1.7 (1.4)
C-reactive protein, mg/L *Mdn* (range)	1.05 (0.14 - 33)	1.10 (0.14 - 16)
C-reactive protein < 10, mg/L *Mdn* (range)	0.93 (0.14 - 7.2)	1.00 (0.14 - 6.8)

Note. LDL = low-density lipoprotein cholesterol. HDL = high-density lipoprotein cholesterol.

[a] Values in mmol/L for ratios. * $p < .05$.
